# Factors associated with stunting among children below five years of age in Zambia: evidence from the 2014 Zambia demographic and health survey

**DOI:** 10.1186/s40795-018-0260-9

**Published:** 2018-12-20

**Authors:** Bubile Mzumara, Phoebe Bwembya, Hikabasa Halwiindi, Raider Mugode, Jeremiah Banda

**Affiliations:** 1 University of Zambia School of Public Health, Nationalist Road, U. T. H, P.O. Box 50110, Lusaka, Zambia; 2 University of Zambia, School of Public Health, Nationalist Road, U .T. H, P.O. Box 50110, Lusaka, Zambia; 3 National Food and Nutrition Commission, Plot#5112 Lumumba Road, P. O Box 32669, Lusaka, Zambia

**Keywords:** Stunting, Zambia demographic and health survey, Socio-demographic factors, Children

## Abstract

**Background:**

Stunting continues to be a major public health problem globally. Stunting is a manifestation of many factors including inadequate food intake and poor health conditions. However, poor quality nutritional diets during pregnancy, infancy and early childhood lead to inadequate nutrient intake. The prevalence of stunting in Zambia has been over 40% and remains unacceptably high. There is limited information on factors associated with stunting in Zambia. Thus to better understand factors contributing to the high stunting levels, the 2013/14 Zambia Demographic and Health Survey (ZDHS) data was analysed.

**Methods:**

Data was extracted using a data extraction tool and analysed using Stata version 13. Sample data of 12, 328 children aged 0–59 months was analysed. The analysis involved simple and multiple logistic regression to find associations between independent variables and stunting.

**Results:**

The prevalence of stunting among under five children in Zambia is 40%. From the 4937 children who were stunted, stunting was higher among male children as compared to female children (42.4 and 37.6% respectively). Additional analysis revealed that children whose source of drinking water was improved (33.7%) were less likely to be stunted compared to children whose source of drinking water was poor (47.7%). Stunting was associated with sex and age of a child; mother’s age and education; residence; wealth and duration of breastfeeding. For instance, children whose mothers had higher education showed a 75% reduction of odds compared to children whose mothers had no education (AOR = 0.35, 95%CI: 0.22, 0.54; *p* < 0.05). Similarly, wealth status showed an inverse relationship. Children who came from rich households showed a 32% reduction of odds compared to children who came from poor households (AOR = 0.68, 95%CI: 0.57, 0.82; *p* < 0.05).

**Conclusion:**

The study established that the major predictors of stunting among children under 5 years old in Zambia were sex and age of the child; mother’s age and level of education; wealth status; improved source of drinking water; duration of breastfeeding and residence. Therefore, multiple measures targeted at reducing child stunting should be taken in a bid to influence policy and conceiving of programmes.

## Background

Stunting continues to be a major public health problem globally. A child is ‘stunted’ if his or her height is less than negative two standard deviations below the World Health Organization standard [1]. Globally, about 161 million children under the age of 5 years are stunted while 51 million children do not weigh enough for their height (wasted), and are not in healthy state [2]. Over the years, the prevalence of stunting has reduced but overall progress is insufficient and many children are still at risk. The prevalence of stunting is highest in developing countries, that is, in Asia and Africa where stunting is more prevalent than underweight or wasting. According to UNICEF [3], 40% of children in Eastern and Southern Africa, under 5 years of age are stunted. Stunting has not spared Zambia. According to the recent Zambia Demographic and Health Survey (ZDHS), about 40% of children under-age five are stunted with Northern Province having the highest prevalence at 49% [4].

Long-term restriction of a child’s potential growth brought about by insufficient nutritious food intake coupled with poor health conditions can lead to stunting. Stunting may start as early in pregnancy, infancy and early childhood due to poor quality nutritional diets that may lead to inadequate nutrient intake. Furthermore, poor socio-economic conditions and increased risk of frequent exposure to certain conditions, such as illness or inappropriate feeding practices may give rise to high levels of stunting [5]. Adequate intake of nutrients is, therefore, essential for growth and mental development and long-term good health.

Consequences of stunting include short adult height, effect on health and negative impact on economic development over time [6]. Stunting has long-term effects on individuals and societies; including diminished cognitive and physical development reduced productive capacity and poor health, and an increased risk of degenerative diseases such as diabetes [3]. For instance, short adult height among women has an impact on the health and survival of their children while for men it may even result in low economic productivity. The government of the Republic of Zambia has put in place measures to address the high levels of stunting through child nutrition programmes and Policies such as the National Food and Nutrition Strategic Plan. Some nutrition programmes include infant and young child feeding, management of acute malnutrition, micronutrient deficiency control and hygiene, water and sanitation. However, despite the spelled out commitments and programmes, stunting still remains considerably high among children aged under 5 years in Zambia compared to WHO standards.

In Zambia, information concerning factors associated with stunting has been very scanty and limited. There is, therefore, need to adequately shed more light on why Zambia continues to experience high levels of stunting. The resulting findings would inform policy and programmes. Stunting has a number of implications on children, which later manifests into adulthood and negatively impact the nation as a whole. Thus, the purpose of this study was to identify socio-economic factors that were associated with stunting among children aged 0 to 59 months in Zambia.

## Methods

### Study setting

Zambia is a landlocked country located in Sub-Sahara African. It has 10 provinces namely Central, Copperbelt, Eastern, Luapula, Lusaka, Muchinga, Northern, North-Western, Southern and Western. These provinces are further sub-divided into districts, constituencies and wards. Economically the country mostly depends on Copper and Cobalt exports. The majority of the population lives in the rural areas and are highly dependent on agriculture for their livelihood. Zambia like most developing countries has a young population with 45.4% of persons aged below 15 years with a life expectancy at birth estimated at 51.2 years (51.7 years in rural areas and 50.8 years in urban areas) [4].

### Data sources

The study extracted data for children less than 5 years of age from the 2013/2014 Zambia Demographic Health Survey database.. The ZDHS was a cross-sectional survey which gathered information on levels and trends in fertility, childhood mortality, use of family planning methods, and maternal and child health indicators including HIV and AIDS [4]. The survey was a nationally representative probability sample of women in the reproductive age 15 to 49 and men 15 to 59 years. The ZDHS provides information on levels and trends in fertility, childhood mortality, use of family planning methods, and maternal and child health indicators including HIV and AIDS at national level for both rural and urban areas of the country [4].

### Sampling and data collection methods

The ZDHS adopted frame of primary sampling units of the 2010 Zambian Census of Population and Housing. The sampling frame consisted of 25,631 Enumeration areas (EAs) and 2,815,897 households. The 2013 /2014, ZDHS adopted a two-stage stratified–cluster sample design where enumeration areas (EAs) where the first stage units and households were selected during the second stage units. The study only included data on children aged zero to 59 months of age with age and height measurements. An enumeration area is a convenient geographical area with an average size of 130 households or 600 people. The sample consisted of 722 Standard enumeration areas (SEAs) from which a selection of 18,050 households was made. The survey interviewed all women aged 15–49 and men aged 15–59 who were either permanent residents of the households or visitors present in the households on the night before the survey. The ZDHS collected data using three questionnaires, namely, the Household Questionnaire, the Woman’s Questionnaire, and the Man’s Questionnaire. The survey interviewed 16,411 women aged 15–49 and 1, 4773 men age 15–59. Children aged 0–59 months and women aged 15–49 who were usual residents of or visitors in the household had their height and weight measured to assess nutritional status. The survey captured 13,554 children under-five, however only 12, 328 children had their height and weight measurements taken.

### Statistical analysis

The main objective of the study was to identify factors that were associated with stunting in Zambia. The study adopted the United Nations Children’s Fund (UNICEF) conceptual framework of the determinants of the nutritional status as it represented a comprehensive aspects of how undernutrition is the outcome of specific development problems related directly to the dietary intake and the health status of an individual. The ZDHS dataset was de-identified to prevent a person’s identity from being linked with information. Firstly, a binary variable was created to define stunting that is “stunted” (Z-score equal to and less than − 2 SD) and “not stunted” (Z-score greater than − 2 SD). Based on literature and guidance from the conceptual framework the study included the following independent variables: sex of child, age of the child, mother’s Education, age of Mother, number of children under-five in the household, duration of breastfeeding, residence (Urban/Rural), place of delivery, type of delivery assistance, wealth status, and quality of source of drinking water. In addition, a variable was created and categorized as either being improved (piped water, protected well and spring, bottled water and rainwater) and non-improved (unprotected well and spring, tanker, surface water and other). With regards to wealth index used in the ZDHS it was a measure that has been used in many DHS and other country-level surveys to indicate inequalities in household characteristics, use of health care and other services, and health outcomes [4]. The indicator serves as a level of wealth that is consistent with expenditure and income measures. It is constructed using household asset data following a principal components analysis [ibid].

Statistical analysis was performed using Stata version 13 (StataCorp, College Station, Texas, USA). The datasets for women and households were used and applied sample weights to adjust for nonresponse. The data assumed normal distribution, as the sample size was relatively big and representative of the population. The main analysis involved descriptive statistics such as proportions to summarise characteristics of participants. Pearson’s Chi-Square test was used to explore relationships between prevalence of stunting and the independent variables. Reported *p*-values< 0.05 and 95% standard normal level were considered statistically significant that is suggesting strong evidence of an association between stunting and an independent variable. In addition, simple and multiple logistic regression was done to measure the net associations of all variables and stunting. Results were reported for each factor and prevalence of stunting after which predictors with significant *p*-values (that is *p* < 0.05 and 95% confidence interval) were considered for multiple logistic analysis. In the multiple regression, the final model was generated using a researcher led backward stepwise regression. Variables were entered in the model and associations were considered statistically significant when *p* < 0.05. The Odds ratios determined the odds of being stunted or not.

### Ethical consideration

Institutional authorization was obtained from the University of Zambia Biomedical Research Ethics Committee (UNZABREC) to conduct the research (Reference number 009–06-16). The Central Statistical Office (CSO) granted permission to use the 2013/14 ZDHS dataset for the study. The researcher ensured unauthorized access, accidental loss or destruction of the dataset by encrypting the dataset on a computer accessible using a password.

## Results

**Fig. 1:**
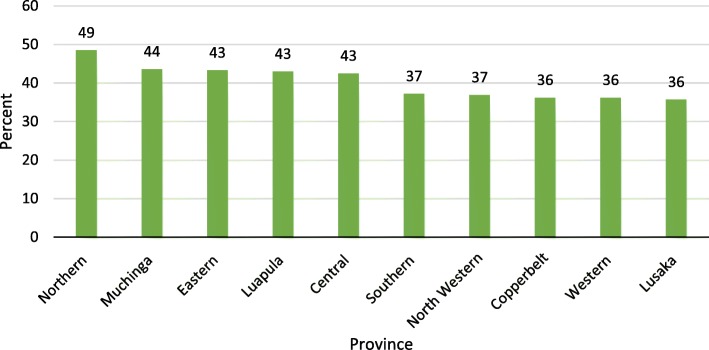
Percentage distribution of Stunting by province

The study included 12,328 children aged 0–59 months who had their height and age measured. The results showed that 4937 (40%) children under 5 years of age were stunted. Northern Province had the highest prevalence of stunting while Lusaka province had the lowest. Figure 1 below shows prevalence of stunting across the country by province.

Prevalence of stunting was higher among male children compared to female children at 42 and 38% respectively. Lowest prevalence rates of stunting included the following attributes: children whose mothers had higher education, children in urban areas, children from a rich status, children exposed to improved source of drinking water, delivered from private mission hospital, mothers received delivery assistance from a health professional, one or two under five children in the H/H, children aged below 6 months. See Table 1 below presents the descriptive statistics of stunting and independent variables.

**Table 1:** Prevalence of stunting in children under five by characteristics of children (*n* = 4937)

Characteristics	Children with stunting	Children without stunting	*p*- value
Sex of Child			
Male	2626 (42.4)	3562 (57.6)	< 0.001
Female	2311 (37.6)	3828 (62.4)	
Age of Mother (years)			
15–19	343 (43.9)	438 (56.1)	0.146
20–24	1200 (41.8)	1531 (58.2)	
25–29	1154 (39.4)	1777 (60.6)	
30–34	944 (39.4)	1453 (60.6)	
35–39	601 (37.9)	984 (62.1)	
40–44	308 (38.5)	491 (61.5)	
45–49	65 (35.1)	119 (64.9)	
Mother’s Level of Education			
No Education	585 (44.1)	725 (55.4)	< 0.001
Primary (grade 1–7)	2738 (42.0)	3780 (58.0)	
Secondary (grade 8–12)	1230 (36.9)	2104 (63.1)	
Higher (college or university)	74 (18.1)	333 (81.9)	
Residence			
Urban	1491 (36.0)	2649 (64.0)	< 0.001
Rural	3447 (42.1)	4741 (57.9)	
Wealth Status			
Poor	2566 (44.6)	3192 (55.4)	< 0.001
Middle	1047 (40.2)	1557 (59.8)	
Rich	1325 (33.4)	2641 (66.6)	
Source of Drinking Water			
Improved	2459 (37)	4125 (63)	< 0.001
Non-improved	1958 (44)	2519 (56)	
Place of Delivery			
Home	1572 (44.1)	1996 (55.9)	< 0.001
Government/public sector	2687 (38)	4391 (62.0)	
Private mission Hospital	196 (36.8)	336 (63.2)	
Other	49 (44.0)	603 (56.0)	
Type of delivery assistance			
Health Professional	2726 (37.7)	4507 (62.3)	< 0.001
TBA	842 (44.1)	1068 (55.9)	
Relative or Other	762(43.5)	989 (56.5)	
No One	170 (45.1)	207 (54.9)	
Duration of Breastfeeding (months)			
0–6	78 (47.1)	87 (52.9)	< 0.001
7–12	212 (40.2)	315 (59.8)	
13–18	955 (40.9)	1381 (59.1)	
19–47	1711 (43.3)	2242 (56.7)	
Never Breast fed	1777 (43.3)	2324 (56.7)	
Still Breastfeeding	1415 (35.0)	2622 (65.0)	
U5 children in H/H			
1 or 2	3794 (39.8)	5741 (60.2)	0.3937
3–5	1060 (40.6)	1550 (59.4)	
6+	33 (53.3)	29 (46.7)	
Child’s age (months)			
< 6	141(13.6)	891(86.4)	< 0.001
6–11	380(31.9)	811(68.1)	
13–23	1212(48.4)	1294 (51.6)	
24–35	1249(51)	1201(49)	
36–47	1038(41.6)	1459(58.4)	
48–59	919 (34.6)	1734(65.4)	

Results from the simple and multiple logistic regression analysis are presented in Tables 2 and 3. The adjusted odds ratio shows that sex of a child, age of a child, residence, mothers’ level of education, wealth status, mothers’ age, duration of breastfeeding and improved source of drinking water were among the factors associated with stunting in children. The associations between stunting and the variables were considered to be statistically significant when *p* < 0.05. Table 2 below shows results from the simple and multiple logistic regression.

**Table 2:** Crude and adjusted measures of the effect of independent variables on stunting in children aged 0–59 months

Characteristics	Crude OR (95%CI)	Adjusted OR (95%CI)
Sex of Child		
Male	1	1
Female	0.85 (0.78–0.92)*	0.80 (0.73, 0.88)*
Age of Mother (years)		
15–19	1	1
20–24	0.95 (0.79–1.12)	0.83 (0.69–1.00)
25–29	0.85 (0.71–1.02)	0.73 (0.60–0.89)
30–34	0.86 (0.72–1.04)	0.73 (0.60–0.89)
35–39	0.80 (0.66–0.98)*	0.64 (0.52–0.80)*
40–44	0.85 (0.68–1.05)	0.65 (0.51–0.83)*
45–49	0.74 (0.50–1.09)	0.60(0.40–91)
Mother’s Level of Education		
No Education (	1	1
Primary (grade 1–7)	0.93 (0.8–1.08)	0.93(0.79–1.10)
Secondary (grade 8–12)	0.74 (0.63–0.87)*	0.84 (0.70–1.02)
Higher (college and university)	0.26 (0.18–0.38)*	0.35 (0.23–0.55)*
Residence		
Urban	1	1
Rural	1.32 (1.18–1.47)*	0.78 (0.68–0.92)*
Wealth Index		
Poor	1	1
Middle	0.84 (0.75–0.95)*	0.84(0.74–0.96)
Rich	0.61 (0.54–0.69)*	0.69(0.57–0.82)*
Source of Drinking Water		
Improved	1	
Non-improved	1.31 (1.19–1.44)*	1.13 (1.02–1.26)
Place of Delivery		
Home	1	1
Gov public sector	0.77 (0.7–0.85)*	0.88 (0.68–1.15)
Private mission Hospital	0.71 (0.57–0.88)	0.84 (0.60–1.17)
Other	0.96 (0.63–1.47)	0.94 (0.60–1.48)
Type of delivery assistance		
Health Professional	1	1
TBA	1.31 (1.16–1.48)*	0.98 (0.77–1.25)
Relative or Other	1.29 (1.14–1.46)*	0.98 (0.74–1.32)
No One	1.30 (1.03–1.65)	1.04 (0.72–1.51)
Duration of Breastfeeding (months)		
0–6	1	1
7–12	0.75 (0.51–1.12)	0.67 (0.44–1.01)
13–18	0.77 (0.52–1.12)	0.65 (0.44–0.96)
19–47	0.86 (0.59–1.28)	0.69 (0.46–1.04)
Never Breast fed	0.86 (0.59–1.28)	0.97 (0.55–1.70)
Still breastfeeding	0.60 (0.41–0.89)	0.65 (0.42–1.00)
U5 children in H/H		
1 or 2	1	1
3–5	1.04 (0.94–1.17)	1.1 (0.96–1.22)
6+	1.45(0.67–3.13)	1.71(0.79–3.71)
Child age (months)		
< 6	1	1
6–11	2.22 (1.63–3.01)*	3.22 (2.44–4.25)*
13–23	4.31 (3.2–5.8)*	4.52 (5.03–8.84)*
24–35	5.16 (4.04–6.61*)	5.88 (5.26–9.75)*
36–47	7.84 (6.09–10.1)*	8.97 (3.76–7.20)*
48–59	6.85 (5.42–8.65)*	8.3 (2.76–5.18)*

**Table 3:** Predictors of Stunting using Backward Step Multiple Regression

Characteristics	Adjusted Odds Ratio (95%CI)	Adjusted *P*-value
Sex of Child		
Male	1	
Female	0.80 (0.73–0.88)	< 0.001
Age of Mother (years)		
15–19	1	
20–24	0.83 (0.69–1.00)	0.046
25–29	0.74 (0.60–0.90)	0.002
30–34	0.73 (0.60–0.89)	0.002
35–39	0.65 (0.52–0.81)	< 0.001
40–44	0.65 (0.51–0.83)	< 0.001
45–49	0.61 (0.41–0.92)	0.018
Mother’s Level of Education		
No Education	1	
Primary (grade 1–7)	0.93(0.79–1.10)	0.411
Secondary (grade 8–12)	0.83 (0.69–1.00)	0.055
Higher (college and university)	0.35 (0.22–0.54)	< 0.001
Residence		
Urban	1	
Rural	0.81 (0.70–0.95)	0.009
Wealth Index		
Poor	1	
Middle	0.85(0.74–0.97)	0.013
Rich	0.68(0.57–0.82)	< 0.001
Source of Drinking Water		
Improved	1	
Non-improved	1.13 (1.03–1.27)	0.016
Duration of Breastfeeding (months)		
0–6	1	
7–12	0.66 (0.44–1.01)	0.054
13–18	0.65 (0.44–0.97)	0.034
19–47	0.69 (0.46–1.04)	0.075
Never BF	0.96 (0.54–1.69)	0.883
still BF	0.65 (0.42–0.99)	0.045
Child age (months)		
< 6	1	
6–11	3.23(2.46–4.26)	< 0.001
13–23	6.77 (5.24–8.75)	< 0.001
24–35	7.08 (5.2–9.65)	< 0.001
36–47	5.18 (3.74–7.16)	< 0.001
48–59	3.76 (2.74–5.16)	< 0.001

Table 3 summarizes the results of the backward stepwise logistic regression models, which measured the net associations of all the independent variables with stunting. For characteristics that remained significant in the model, the odds ratios did not change substantially from the bivariate analysis. Child’s sex was significant for predicting stunting. Children in rural areas were 19% less likely to be stunted compared with children living in urban areas (AOR = 0.81, 95%CI: 0.70, 0.95; *p* < 0.05), While children aged 24 to 35 months of age had 7 times higher odds of being stunted compared with children less than 6 months. The analysis showed that, child’s sex, child age, mothers’ age and level of education, residence, improved source of drinking water and duration of breastfeeding were significant for predicting stunting.

## Discussion

Early childhood development is crucial especially the first 1000 days of an infant’s life after which stunting is irreversible. Long-term effects of stunting include diminished cognitive and physical development, poor health and adult short stature. The study intended to identify the factors associated with stunting among children aged 0 to 59 months using latest ZDHS data. The prevalence of stunting among children under five was 40% and was more prevalent among male children than female children. This finding is similar to other studies conducted around the world that have also documented higher prevalence of stunting in boys than in girls [7–9]. According to Chirande et al. [5] sex differences could also be attributed to behavioural patterns of communities for instance, favouritism towards daughters. Additionally, epidemiological evidence depicts boys to be biologically more vulnerable to morbidity [10, 11].

Over the years studies have observed that populations that live in rural areas have been more susceptible to nutritional deficits due to a number of disadvantages however, according to the results of the present study, children in urban areas had a higher risk for stunting than children in rural areas. Our study agrees with other studies that found similar results [12, 13]. This risk of stunting in urban areas might be due to decreased maternal contact time due to work schedules of working mothers that may bring about short period of breastfeeding, early cessation of breastfeeding and improper complementary food, which have a largely negative effect on the growth of the children. This entails that most of the urban population might actually be urban poor who live in informal settlements/unauthorised slums in abject poverty, poor water and sanitation, high food insecurity and limited nutritious foods. Rural populations may have opportunities to grow nutritious rich foods while urban poor are highly dependent on food purchase and diets lack diversity. On the contrary, our study contradicts other studies that have revealed no significant association between urban or rural location [14, 15]. There is, therefore, need of establishing strategies that facilitate proper nutrition and child health in urban areas especially among the poor.

Mother’s education continues to be associated with stunting. Higher odds of stunting being observed among children whose mothers had no education. The same has been confirmed in previous studies and the results imply that maternal education may provide protective effects against all under-nutrition indicators in children [5, 14, 16, 17]. Mothers who are more educated are more likely to be more conscious about their children’s health. Moreover, due to exposure to media they are likely to have better child and healthcare knowledge of nutrition leading to better feeding practices. However, other studies show contrary results in that there was no significant association between stunting and maternal education [18, 19]. While most studies have shown that maternal education is a determinant of a child’s nutritional status, other studies have actually indicated that father’s education is equally an important factor for child nutrition [20, 21]. Therefore, the importance of maternal education might vary from country to country and the difference may be probably due to differences in study design and as well as different socio-economic statuses of countries.

In relation to mother’s age, the differences in prevalence of stunting decreased with maternal age. Lower odds of stunted were observed among children whose mothers were aged above 35 years old. The study results corroborate with other studies [5, 22]. This may be because young mothers require adequate nutrition for them to grow into adults and as a young mother; food shared in small proportion between the infant and the mother is not adequate. In addition, younger mothers may tend to have poor knowledge and practices of good nutrition for young children.

Water and sanitation also has a significant impact on child nutritional status as lack of water in households makes basic hygiene somewhat unattainable. In the present study, stunting was significantly associated with improved source of drinking water. Children whose source of drinking water was non-improved were likely to be stunted compared to children whose source of water was improved. This may be attributed to the fact that non-improved water sources may be contaminated and thus may increase risk of infection such as diarrhoea. The study findings are consistent with other studies [23–26]. However, the study findings are also contrary to previous studies [16, 18, 27, 28], that depict no significant association between source of drinking water and stunting.

In relation to number of children in the household, smaller families are generally socio-economically advantaged accompanied by improved quality of life. In the present study, although the highest OR for stunting correspond to children belonging to families with six or more children the same was not confirmed in the multiple analysis after adjustment by other factors. These results are contrary to other studies [9, 22, 29, 30], which observed a significant relationship between stunting and number of under five children in a household.

Type of delivery assistance and place of delivery showed significant statistical relations in the bivariate analysis, however, with respect to multiple logistic regression analysis they did not show statistical significance. In this study type of delivery assistance and place of delivery were, therefore, statistically insignificant factors with regard to the association with stunting.

The present study had a number of limitations. Firstly, an in-built limitation of cross-sectional data is their snapshot nature that makes establishing a temporal sequence of events and drawing causal inferences difficult as this pertains to the period and season the survey was undertaken. A few key variables could not be included because of difference in classifications, as data was collected and classified by ZDHS team. For instance, the ZDHS only classifies urban residence as urban even though it includes peri-urban areas thus the definition was not precise. Although the study excluded other key variables from the analysis, our model did reflect all those variables linked to childhood stunting. The ZDHS often has a delay in publishing results, which implies that information might not be a true reflection of the current situation.

## Conclusions

Stunting is associated with factors including nutrition, socio-economic, demographic and environmental factors that impact on children’s health and wellbeing. These factors often not only influence stunting but also influence nutritional status of a child. Therefore, improvements in maternal education, household wealth, diet diversity, water and sanitation could improve health status of children. Stunting affects health status and productivity later in adult life. Thus, the consequences of socio-economic inequalities in childhood nutritional status are likely to be recurring. Therefore, with this information on factors associated with stunting, there is need to address all causes of stunting in an integrated manner. Policies and programmes should also give greater attention to improving maternal education especially among younger mothers, improve social-economic status and improve water sources, sanitation and hygienic practices.

## Abbreviations

CSO: Central statistics office
HAZ: Height-for-Age Z-score
SUN: Scaling up Nutrition
UN: United Nations
UNICEF: United Nation International Children Fund
UNZA: University of Zambia
WHO: World Health Organization
ZDHS: Zambia Demographic Health Survey
